# From data to value in multimodal learning

**DOI:** 10.1016/j.patter.2026.101615

**Published:** 2026-08-14

**Authors:** Chunlei Tang, Xing Jia, Yun Xiong

**Affiliations:** 1 DataLattice Research, Boston, MA, USA; 2 Harvard Medical School, Boston, MA, USA; 3 Shanghai Key Laboratory of Data Science, School of Computer Science, Fudan University, Shanghai, China; 4 School of Computer Science and Technology, Donghua University, Shanghai, China

## Abstract

Multimodal learning enables the integration of heterogeneous data, but value emerges only when data are transformed into stable, reusable evidence. Such evidence can reduce interpretive cost, support trust across contexts, and accumulate into data capital.

## Main text

Health care operates as a fundamentally multimodal system. Clinical decisions depend on heterogeneous sources of information: clinical narratives, radiology images, laboratory measurements, physiological signals, genomic profiles, and, increasingly, behavioral and environmental data collected over time.^1^ These data are produced in different settings, by different actors, and under different conditions. They do not naturally align. What exists in practice is not a unified dataset but a fragmented evidence landscape that must be continually reconstructed.

In everyday clinical work, this reconstruction is not abstract. A single diagnosis may require the integration of imaging findings, laboratory trends, prior notes, and evolving symptoms. These sources often disagree, arrive at different times, or carry different levels of reliability. Clinicians resolve these inconsistencies through judgment, experience, and repeated verification. The process is labor intensive and inherently contextual, consistent with long-standing models of human interaction with complex systems.^2^ Multimodal systems are expected to reduce this burden. In many cases, they add to it. Healthcare therefore illustrates a broader challenge: heterogeneous data alone are not sufficient unless they can be transformed into stable evidence that supports trust and reuse.

Multimodal learning is often presented as a natural response to this reality and has been widely studied as a framework for integrating heterogeneous data across modalities.^3^^,^^4^ It also reflects a deeper ambition. Integrating heterogeneous data is an attempt to make information usable across contexts, to move from isolated observations toward something that can be accumulated and reused. In our prior work on clinical data systems and evidence-centered data infrastructures, we observed that increasing data heterogeneity often raises interpretive burden rather than improving decision reliability.^5^ Yet, the outcomes have been uneven. As more modalities are introduced, systems become harder to interpret, more costly to maintain, and less aligned with clinical workflows. More data do not reliably translate into more value.

This contrast is visible in clinical adoption. Systems built on a single modality, especially text, are more likely to be used. They fit existing workflows. They require less coordination across data sources. They are easier to interpret and to validate. Their success is not a function of representational power alone but of lower interpretive burden. In many settings, simpler systems produce more usable results because they reduce the effort required to make sense of the output.

The difficulty is not simply that multimodal data are different. It is that they are different in incompatible ways. Laboratory values are structured and standardized. Clinical narratives are unstructured and context dependent. Imaging data are high dimensional and often ambiguous. Physiological signals evolve over time. These differences reflect distinct measurement processes, clinical meanings, and temporal scales. Bringing them together does not just increase information. It expands the number of relationships that must be interpreted, reconciled, and trusted.

In practice, this expansion has concrete consequences. Multimodal systems often require extensive data preprocessing, alignment across modalities, and manual curation to ensure consistency. Missing data, temporal misalignment, and conflicting signals are common. Even in well-studied settings such as image-to-text integration in radiology, ensuring factual consistency and completeness remains challenging.^6^^,^^7^ Even after model development, outputs must be reviewed and contextualized before use. In some settings, this leads to parallel workflows in which automated outputs are checked against traditional interpretations, effectively duplicating effort rather than reducing it. The system does not replace work. It reorganizes and often amplifies it.

Explainability is often proposed as a solution. In multimodal systems, it rarely resolves the problem. Explanations inherit the same heterogeneity as the data. Different modalities support different interpretations. A model may be internally coherent within each modality while remaining difficult to reconcile across them. The burden shifts to the user, who must interpret not only the result but also the relationships among modalities. The result is not clarity but additional work, even under human-centered artificial intelligence (AI) frameworks that emphasize reliability and interpretability.^8^ This shift of burden points to a more fundamental constraint.

### The limitation is not technical; it is economic

**Figure 1:**
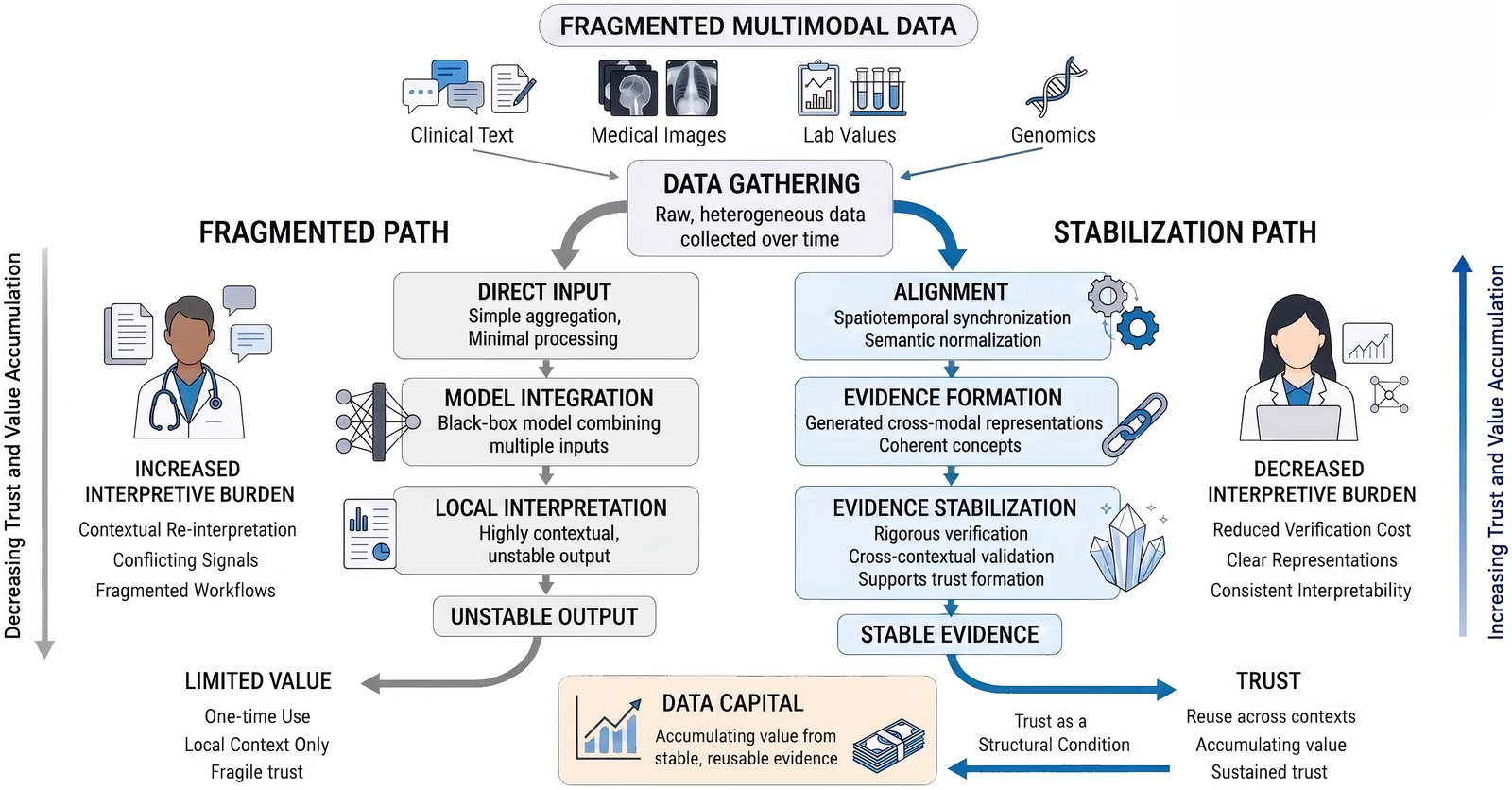
The path from fragmented multimodal data to data capital Multimodal data do not automatically generate value. The figure illustrates two possible trajectories. In the fragmented path, heterogeneous data are aggregated with limited integration, increasing interpretive burden and producing unstable outputs. In the stabilization path, alignment, evidence formation, and evidence stabilization transform heterogeneous inputs into stable evidence. Under these conditions, trust emerges as a structural condition that enables the reuse, accumulation, and amplification of value across contexts, allowing data to function as a form of capital. Google Gemini was used to assist with graphical refinement and visual rendering of certain elements. The conceptual framework, scientific content, interpretation, and final figure design were developed and validated by the authors.

What is missing is not more data but a more reliable way to turn data into evidence. As illustrated in Figure 1, practical value emerges not from the accumulation of modalities alone but from the stabilization of evidence that supports trust and reuse. The production of evidence is inseparable from the production of data, unfolding through processes that are often fragmented, iterative, and context dependent. In practice, evidence does not emerge fully formed but is gradually stabilized through stages of data generation, alignment, and verification before it can support consistent interpretation. Rather than being an abstract property, trust emerges as a structural condition when heterogeneous data are crystallized into stable evidence.

This progression can be observed in practical multimodal applications. In radiology report generation, for example, imaging data and textual descriptions must be aligned to produce clinically meaningful outputs. Although recent systems have achieved impressive technical performance, ensuring factual consistency, completeness, and contextual reliability remains challenging.^6^^,^^7^^,^^8^ Outputs often require human review against the original images before they can be trusted and reused in clinical workflows. The challenge is therefore not simply one of multimodal integration but of transforming heterogeneous data into stable evidence that can support consistent interpretation across contexts.

This cost is not evenly distributed. It falls on clinicians, data engineers, and institutions that must absorb the effort required to make systems usable. In resource-constrained settings, this burden becomes a barrier to adoption. In more advanced settings, it leads to selective use, where only parts of a multimodal system are trusted or routinely applied. In both cases, the gap between technical capability and practical value persists.

This transformation follows a progression. Raw data are first aligned across modalities, enabling comparison. From there, evidence can be formed to support consistent interpretation rather than isolated readings. Only when this evidence becomes stable can it be reused across contexts. At each step, the burden of interpretation is reduced and the meaning of outputs becomes more consistent.

Without this progression, multimodal outputs remain fragile. They depend on context and must be reinterpreted each time they are used. They do not accumulate. When evidence is stabilized, it behaves differently: it can move across settings, persist over time, and support consistent interpretation among different users. Decisions can then build on prior results rather than restarting from scratch. In this sense, trust is not added at the end but emerges from the stabilization of evidence within the system.

This also clarifies how value emerges. Unstable outputs generate work. They require repeated interpretation and verification. Their use is local and temporary. Stable evidence reduces that burden. It supports reuse. It allows decisions to extend beyond a single encounter. In clinical practice, this may take the form of standardized interpretations that can be applied across patient populations or validated outputs that reduce the need for repeated review. Under these conditions, value can be preserved, reused, and further enhanced rather than repeatedly recreated. Data begin to function as capital: cumulative rather than disposable, transferable rather than local, and capable of generating value beyond a single use.

The integration of AI with multimodal data is often framed as a question of capability.^9^^,^^10^^,^^11^ The harder question is how to make such systems usable. Increasing model complexity does not resolve this. What matters is whether heterogeneous data can be organized into evidence that remains stable across contexts. Without that stability, more data increases uncertainty. With it, data can support trust, coordination, and repeated use.

Multimodal learning is therefore more than a technical advance. It is an attempt to reorganize how data function within complex systems. The challenge is not to integrate more modalities but to establish the conditions under which data can be crystallized into stable evidence. Only under these conditions can trust be sustained. Trust then becomes a structural condition for reuse, enabling value not only to persist but also to generate additional value through repeated use over time. In this sense, data capital is not created through the collection of more data but through the stabilization of evidence that allows value to be preserved, enhanced, accumulated, and reused across contexts.

## Acknowledgments

This work was supported by the Fundamental Research Funds for the Central Universities (no. 2232025D-34) and the Noncommunicable Chronic Diseases – National Science and Technology Major Project (nos. 2024ZD0532400 and 2024ZD0532403).

## Declaration of interests

The authors declare no competing interests.
